# *N*-(2,4-Dioxo-1,3-thia­zolidin-3-yl)-2-(4-isobutyl­phen­yl)propanamide

**DOI:** 10.1107/S160053680903027X

**Published:** 2009-08-08

**Authors:** Hoong-Kun Fun, Jia Hao Goh, A. C. Vinayaka, B. Kalluraya

**Affiliations:** a X-ray Crystallography Unit, School of Physics, Universiti Sains Malaysia, 11800 USM, Penang, Malaysia; b Department of Studies in Chemistry, Mangalore University, Mangalagangotri, Mangalore 574 199, India

## Abstract

In the title compound, C_16_H_20_N_2_O_3_S, the thia­zolidine ring is approximately planar [maximum deviation = 0.020 (2) Å] and forms a dihedral angle of 86.20 (11)° with the benzene ring. The mean plane through the propanamide unit forms dihedral angles of 88.54 (12) and 76.36 (12)°, respectively, with the thia­zolidine and benzene rings. In the crystal structure, mol­ecules are linked into chains along the *a* axis by N—H⋯O inter­actions. These chains are inter­connected into two-dimensional arrays parallel to the *ab* plane by three different C—H⋯O inter­actions. The crystal structure is further stabilized by weak inter­molecular C—H⋯π and N⋯O [2.713 (2) Å] inter­actions.

## Related literature

For general background to the synthesis, pharmacological properties and applications of compounds incorporating ibuprofen, see: Aktay *et al.* (2005 ▶); Palaska *et al.* (2002 ▶); Verma & Saraf (2008 ▶). For related structures, see: Fun *et al.* (2009*a* ▶,*b* ▶). For the stability of the temperature controller used for the data collection, see: Cosier & Glazer (1986 ▶).
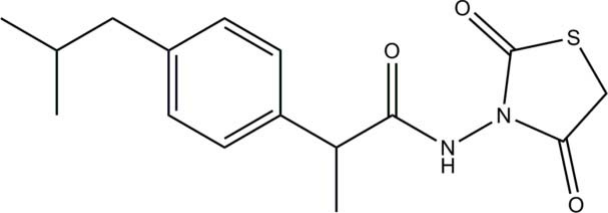


## Experimental

### 

#### Crystal data

C_16_H_20_N_2_O_3_S
*M* *_r_* = 320.40
Orthorhombic, 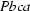

*a* = 9.7305 (1) Å
*b* = 11.3991 (2) Å
*c* = 29.6323 (4) Å
*V* = 3286.78 (8) Å^3^
*Z* = 8
Mo *K*α radiation
μ = 0.21 mm^−1^
*T* = 100 K
0.24 × 0.21 × 0.09 mm

#### Data collection

Bruker SMART APEXII CCD area-detector diffractometer
Absorption correction: multi-scan (**SADABS**; Bruker, 2005 ▶) *T*_min_ = 0.951, *T*_max_ = 0.981
33569 measured reflections
3783 independent reflections
2927 reflections with *I* > 2σ(*I*)
*R*_int_ = 0.079

#### Refinement

*R*[*F* ^2^ > 2σ(*F* ^2^)] = 0.064
*wR*(*F* ^2^) = 0.143
*S* = 1.20
3783 reflections
279 parameters
H atoms treated by a mixture of independent and constrained refinement
Δρ_max_ = 0.44 e Å^−3^
Δρ_min_ = −0.26 e Å^−3^

### 

Data collection: *APEX2* (Bruker, 2005 ▶); cell refinement: *SAINT* (Bruker, 2005 ▶); data reduction: *SAINT*; program(s) used to solve structure: *SHELXTL* (Sheldrick, 2008 ▶); program(s) used to refine structure: *SHELXTL*; molecular graphics: *SHELXTL*; software used to prepare material for publication: *SHELXTL* and *PLATON* (Spek, 2009 ▶).

## Supplementary Material

Crystal structure: contains datablocks global, I. DOI: 10.1107/S160053680903027X/tk2517sup1.cif

Structure factors: contains datablocks I. DOI: 10.1107/S160053680903027X/tk2517Isup2.hkl

Additional supplementary materials:  crystallographic information; 3D view; checkCIF report

## Figures and Tables

**Table 1:** Hydrogen-bond geometry (Å, °)

*D*—H⋯*A*	*D*—H	H⋯*A*	*D*⋯*A*	*D*—H⋯*A*
N2—H1*N*2⋯O3^i^	0.85 (3)	1.94 (3)	2.713 (2)	151 (2)
C2—H2*A*⋯O1^ii^	0.89 (3)	2.53 (3)	3.209 (3)	133 (3)
C2—H2*B*⋯O2^iii^	0.92 (3)	2.45 (3)	3.371 (3)	174 (2)
C5—H5⋯O1^iv^	1.02 (3)	2.46 (2)	3.190 (3)	127.6 (18)
C2—H2*A*⋯*Cg*2^ii^	0.90 (3)	2.99 (3)	3.474 (3)	116 (2)

Symmetry codes: (i) 
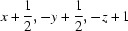
; (ii) 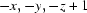; (iii) 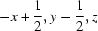; (iv) 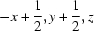.  *Cg*2 is the centroid of the C6–C11 ring ring.

**Fig. 1.**
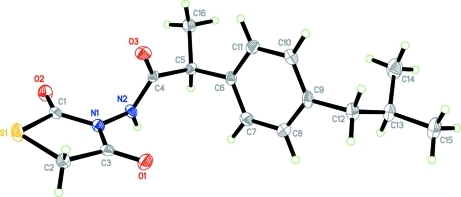
The molecular structure of (I), showing 50% probability displacement ellipsoids and the atom-numbering scheme.

**Fig. 2.**
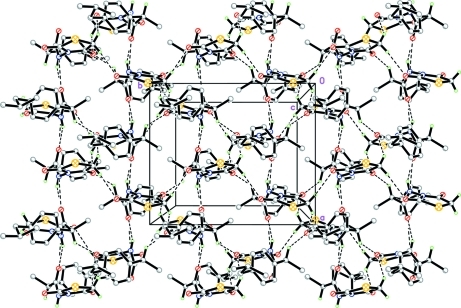
2-D arrays parallel to the ab plane in (I), viewed along the c axis. H atoms not involved in intermolecular interactions (dashed lines) have been omitted for clarity.

## supplementary crystallographic information

### Comment

The synthesis of compounds incorporating ibuprofen has been attracting widespread attention due to their diverse pharmacological properties, such as anti-microbial, anti-inflammatory, analgesic and anti-tumor activities (Palaska *et al.*, 2002; Aktay *et al.*, 2005). There are numerous biologically active molecules with five-membered rings, containing two heteroatoms. For example, thiazolidine is an important scaffold known to be associated with several biological activities (Verma & Saraf, 2008). In view of the above, the synthesis of a new series of 2,4-dioxo-1,3-thiazolidins containing ibuprofen was undertaken. We report here one of these crystal structures, (I).

In (I), Fig. 1, the thiazolidine ring (C1-C3/N1/S1) is approximately planar, with a maximum deviation of 0.020 (2) Å for atom C3. The thiazolidine ring is almost perpendicular to the C6-C12 benzene ring, forming a dihedral angle of 86.20 (11)°. The mean plane through the propanamide (C4/C5/N2/O3) forms dihedral angles of 88.54 (12)° and 76.36 (12)°, respectively, with the thiazolidine and benzene rings. The bond lengths are comparable to those found in closely related structures (Fun *at al.*, 2009a,b).

In the crystal structure (Fig. 2), the molecules are linked into chains along the *a* axis by N2—H1N2···O3 interactions (Table 1). These chains are interconnected into 2-D arrays parallel to the *ab* plane by additional C2—H2A···O1, C2—H2B···O2 and C5—H5···O1 interactions (Table 1). The crystal structure is further stabilized by short N2···O3 contacts of 2.713 (2) Å [symmetry code: -1/2+x, 1/2-y, 1-z] and by weak C2—H2A···Cg2 interactions (Table 1).

### Experimental

Compound (I) was obtained by refluxing 4-amino-5-[1-(4-isobutylphenyl)ethyl]-4*H*-1,3,4-oxadiazole-2-thiol (0.005 mol) and ethylchloroacetate (0.005 mol) in a solution comprising ethanol (20 ml) and pyridine (2 ml) in an oil bath for 8 h. The solid product obtained was collected by filtration, washed with ethanol and dried. It was then recrystallized using ethanol. Single crystals were obtained by slow evaporation from an ethanol solution of (I). Yield was 84 %. *M.p.* 466 K.

### Refinement

All H atoms were located from difference Fourier maps and allowed to refine freely [range of C—H = 0.90 (3) - 1.02 (2) Å].

### Crystal data

**Table:**

C_16_H_20_N_2_O_3_S	*F*(000) = 1360
*M**_r_* = 320.40	*D*_x_ = 1.295 Mg m^−^^3^
Orthorhombic, *P**b**c**a*	Mo *K*α radiation, λ = 0.71073 Å
Hall symbol: -P 2ac 2ab	Cell parameters from 5195 reflections
*a* = 9.7305 (1) Å	θ = 2.5–30.0°
*b* = 11.3991 (2) Å	µ = 0.21 mm^−^^1^
*c* = 29.6323 (4) Å	*T* = 100 K
*V* = 3286.78 (8) Å^3^	Plate, colourless
*Z* = 8	0.24 × 0.21 × 0.09 mm

### Data collection

**Table:**

Bruker SMART APEXII CCD area-detector diffractometer	3783 independent reflections
Radiation source: fine-focus sealed tube	2927 reflections with *I* > 2σ(*I*)
graphite	*R*_int_ = 0.079
φ and ω scans	θ_max_ = 27.5°, θ_min_ = 2.5°
Absorption correction: multi-scan (*SADABS*; Bruker, 2005)	*h* = −12→12
*T*_min_ = 0.951, *T*_max_ = 0.981	*k* = −14→13
33569 measured reflections	*l* = −38→36

### Refinement

**Table:**

Refinement on *F*^2^	Primary atom site location: structure-invariant direct methods
Least-squares matrix: full	Secondary atom site location: difference Fourier map
*R*[*F*^2^ > 2σ(*F*^2^)] = 0.064	Hydrogen site location: inferred from neighbouring sites
*wR*(*F*^2^) = 0.143	H atoms treated by a mixture of independent and constrained refinement
*S* = 1.20	*w* = 1/[σ^2^(*F*_o_^2^) + (0.0661*P*)^2^ + 0.7928*P*] where *P* = (*F*_o_^2^ + 2*F*_c_^2^)/3
3783 reflections	(Δ/σ)_max_ = 0.001
279 parameters	Δρ_max_ = 0.44 e Å^−^^3^
0 restraints	Δρ_min_ = −0.26 e Å^−^^3^

### Special details

**Table:**

Experimental. The crystal was placed in the cold stream of an Oxford Cyrosystems Cobra open-flow nitrogen cryostat (Cosier & Glazer, 1986) operating at 100.0 (1)K.
Geometry. All esds (except the esd in the dihedral angle between two l.s. planes) are estimated using the full covariance matrix. The cell esds are taken into account individually in the estimation of esds in distances, angles and torsion angles; correlations between esds in cell parameters are only used when they are defined by crystal symmetry. An approximate (isotropic) treatment of cell esds is used for estimating esds involving l.s. planes.
Refinement. Refinement of F^2^ against ALL reflections. The weighted R-factor wR and goodness of fit S are based on F^2^, conventional R-factors R are based on F, with F set to zero for negative F^2^. The threshold expression of F^2^ > 2sigma(F^2^) is used only for calculating R-factors(gt) etc. and is not relevant to the choice of reflections for refinement. R-factors based on F^2^ are statistically about twice as large as those based on F, and R- factors based on ALL data will be even larger.

### Fractional atomic coordinates and isotropic or equivalent isotropic displacement parameters (Å^2^)

**Table:**

	*x*	*y*	*z*	*U*_iso_*/*U*_eq_	
S1	0.07397 (7)	0.09512 (5)	0.38158 (2)	0.02357 (19)	
O1	0.15979 (17)	0.01544 (15)	0.50523 (6)	0.0235 (4)	
O2	0.13357 (18)	0.31521 (14)	0.40290 (6)	0.0234 (4)	
O3	−0.03571 (16)	0.26390 (15)	0.51261 (6)	0.0209 (4)	
N1	0.15610 (19)	0.17543 (16)	0.45864 (6)	0.0154 (4)	
N2	0.1867 (2)	0.25414 (17)	0.49260 (6)	0.0164 (4)	
C1	0.1250 (2)	0.2144 (2)	0.41525 (8)	0.0169 (5)	
C2	0.0969 (3)	−0.0098 (2)	0.42684 (8)	0.0193 (5)	
C3	0.1398 (2)	0.0567 (2)	0.46830 (8)	0.0171 (5)	
C4	0.0839 (2)	0.28924 (18)	0.52017 (8)	0.0134 (5)	
C5	0.1292 (2)	0.35908 (19)	0.56150 (8)	0.0154 (5)	
C6	0.1110 (2)	0.27896 (19)	0.60228 (8)	0.0161 (5)	
C7	0.2173 (3)	0.2042 (2)	0.61513 (8)	0.0214 (5)	
C8	0.2021 (3)	0.1278 (2)	0.65112 (9)	0.0231 (6)	
C9	0.0800 (3)	0.1236 (2)	0.67589 (8)	0.0186 (5)	
C10	−0.0259 (3)	0.1977 (2)	0.66277 (8)	0.0199 (5)	
C11	−0.0112 (3)	0.2746 (2)	0.62666 (8)	0.0191 (5)	
C12	0.0669 (3)	0.0436 (2)	0.71635 (8)	0.0225 (5)	
C13	0.1343 (3)	0.0931 (2)	0.75930 (8)	0.0241 (6)	
C14	0.0692 (4)	0.2092 (3)	0.77362 (11)	0.0338 (7)	
C15	0.1251 (4)	0.0031 (3)	0.79731 (10)	0.0343 (7)	
C16	0.0467 (3)	0.4732 (2)	0.56402 (9)	0.0211 (5)	
H2A	0.018 (3)	−0.046 (3)	0.4334 (10)	0.037 (8)*	
H2B	0.166 (3)	−0.062 (2)	0.4197 (9)	0.029 (7)*	
H5	0.231 (3)	0.381 (2)	0.5594 (8)	0.014 (6)*	
H7	0.299 (3)	0.204 (2)	0.5973 (9)	0.027 (7)*	
H8	0.277 (3)	0.074 (2)	0.6603 (9)	0.023 (7)*	
H10	−0.111 (3)	0.198 (2)	0.6778 (8)	0.016 (6)*	
H11	−0.083 (3)	0.326 (3)	0.6170 (9)	0.028 (7)*	
H12A	0.107 (3)	−0.031 (2)	0.7094 (9)	0.023 (7)*	
H12B	−0.031 (3)	0.031 (2)	0.7228 (8)	0.015 (6)*	
H13	0.232 (3)	0.109 (2)	0.7529 (10)	0.034 (8)*	
H14A	0.109 (3)	0.239 (3)	0.7992 (11)	0.037 (8)*	
H14B	−0.031 (3)	0.197 (3)	0.7814 (10)	0.038 (8)*	
H14C	0.075 (3)	0.270 (3)	0.7520 (11)	0.046 (9)*	
H15A	0.167 (3)	0.035 (3)	0.8251 (11)	0.042 (9)*	
H15B	0.029 (3)	−0.021 (3)	0.8030 (10)	0.039 (8)*	
H15C	0.171 (3)	−0.073 (3)	0.7888 (10)	0.036 (8)*	
H16A	−0.054 (3)	0.457 (2)	0.5618 (9)	0.030 (8)*	
H16B	0.078 (3)	0.527 (2)	0.5401 (9)	0.024 (7)*	
H16C	0.063 (3)	0.507 (3)	0.5937 (10)	0.032 (8)*	
H1N2	0.271 (3)	0.270 (2)	0.4968 (9)	0.031 (8)*	

### Atomic displacement parameters (Å^2^)

**Table:**

	*U*^11^	*U*^22^	*U*^33^	*U*^12^	*U*^13^	*U*^23^
S1	0.0388 (4)	0.0173 (3)	0.0146 (3)	−0.0015 (3)	−0.0018 (3)	−0.0025 (2)
O1	0.0259 (10)	0.0239 (9)	0.0207 (9)	0.0021 (7)	−0.0022 (7)	0.0052 (7)
O2	0.0339 (10)	0.0151 (8)	0.0213 (9)	−0.0008 (7)	−0.0007 (8)	0.0013 (7)
O3	0.0120 (8)	0.0254 (9)	0.0254 (10)	−0.0013 (7)	−0.0005 (7)	−0.0031 (7)
N1	0.0155 (10)	0.0149 (9)	0.0157 (10)	−0.0010 (7)	−0.0002 (8)	−0.0018 (8)
N2	0.0140 (11)	0.0208 (10)	0.0144 (10)	−0.0025 (8)	−0.0004 (9)	−0.0047 (8)
C1	0.0174 (12)	0.0183 (12)	0.0150 (12)	0.0025 (9)	0.0029 (10)	−0.0019 (9)
C2	0.0265 (14)	0.0139 (11)	0.0174 (12)	0.0006 (10)	0.0028 (10)	0.0025 (9)
C3	0.0137 (11)	0.0194 (11)	0.0182 (12)	0.0041 (9)	0.0027 (10)	0.0002 (9)
C4	0.0152 (12)	0.0118 (10)	0.0132 (11)	0.0013 (9)	−0.0005 (9)	0.0044 (8)
C5	0.0152 (12)	0.0153 (11)	0.0158 (12)	−0.0012 (9)	0.0000 (10)	0.0026 (9)
C6	0.0212 (12)	0.0144 (11)	0.0128 (11)	−0.0016 (9)	−0.0006 (9)	−0.0016 (9)
C7	0.0200 (13)	0.0241 (12)	0.0200 (13)	0.0020 (10)	0.0044 (11)	0.0015 (10)
C8	0.0250 (14)	0.0236 (13)	0.0208 (13)	0.0063 (10)	−0.0005 (11)	0.0027 (10)
C9	0.0284 (13)	0.0162 (11)	0.0113 (11)	−0.0060 (10)	−0.0025 (10)	−0.0024 (9)
C10	0.0207 (13)	0.0214 (12)	0.0176 (12)	−0.0040 (10)	0.0033 (11)	−0.0030 (10)
C11	0.0204 (13)	0.0182 (11)	0.0186 (13)	0.0010 (10)	−0.0032 (10)	−0.0004 (9)
C12	0.0301 (15)	0.0202 (13)	0.0171 (13)	−0.0034 (11)	0.0014 (11)	0.0002 (10)
C13	0.0297 (15)	0.0264 (13)	0.0163 (13)	−0.0029 (11)	−0.0020 (11)	0.0050 (10)
C14	0.051 (2)	0.0285 (15)	0.0223 (15)	0.0008 (14)	−0.0058 (15)	−0.0052 (13)
C15	0.0472 (19)	0.0352 (16)	0.0205 (15)	−0.0025 (14)	−0.0038 (13)	0.0081 (12)
C16	0.0266 (14)	0.0158 (12)	0.0210 (14)	0.0009 (10)	0.0011 (11)	−0.0008 (10)

### Geometric parameters (Å, °)

**Table:**

S1—C1	1.758 (2)	C8—H8	0.99 (3)
S1—C2	1.811 (2)	C9—C10	1.388 (3)
O1—C3	1.207 (3)	C9—C12	1.511 (3)
O2—C1	1.209 (3)	C10—C11	1.391 (3)
O3—C4	1.220 (3)	C10—H10	0.94 (3)
N1—N2	1.381 (3)	C11—H11	0.96 (3)
N1—C3	1.392 (3)	C12—C13	1.538 (4)
N1—C1	1.394 (3)	C12—H12A	0.96 (3)
N2—C4	1.352 (3)	C12—H12B	0.99 (2)
N2—H1N2	0.85 (3)	C13—C15	1.526 (4)
C2—C3	1.503 (3)	C13—C14	1.527 (4)
C2—H2A	0.90 (3)	C13—H13	0.99 (3)
C2—H2B	0.92 (3)	C14—H14A	0.92 (3)
C4—C5	1.526 (3)	C14—H14B	1.01 (3)
C5—C6	1.525 (3)	C14—H14C	0.95 (3)
C5—C16	1.530 (3)	C15—H15A	0.99 (3)
C5—H5	1.02 (2)	C15—H15B	0.98 (3)
C6—C11	1.392 (3)	C15—H15C	1.01 (3)
C6—C7	1.393 (3)	C16—H16A	1.00 (3)
C7—C8	1.385 (3)	C16—H16B	0.98 (3)
C7—H7	0.95 (3)	C16—H16C	0.97 (3)
C8—C9	1.398 (4)		
			
C1—S1—C2	93.19 (11)	C10—C9—C12	121.8 (2)
N2—N1—C3	120.43 (19)	C8—C9—C12	120.6 (2)
N2—N1—C1	120.79 (18)	C9—C10—C11	121.5 (2)
C3—N1—C1	118.35 (19)	C9—C10—H10	121.4 (15)
C4—N2—N1	118.2 (2)	C11—C10—H10	117.1 (15)
C4—N2—H1N2	124.4 (19)	C10—C11—C6	120.6 (2)
N1—N2—H1N2	116.8 (19)	C10—C11—H11	122.8 (16)
O2—C1—N1	124.6 (2)	C6—C11—H11	116.6 (16)
O2—C1—S1	125.66 (19)	C9—C12—C13	113.6 (2)
N1—C1—S1	109.77 (16)	C9—C12—H12A	109.3 (16)
C3—C2—S1	107.87 (16)	C13—C12—H12A	109.3 (16)
C3—C2—H2A	107.0 (19)	C9—C12—H12B	109.0 (14)
S1—C2—H2A	110.9 (19)	C13—C12—H12B	108.0 (14)
C3—C2—H2B	108.1 (17)	H12A—C12—H12B	108 (2)
S1—C2—H2B	110.2 (18)	C15—C13—C14	110.7 (2)
H2A—C2—H2B	112 (3)	C15—C13—C12	109.9 (2)
O1—C3—N1	123.1 (2)	C14—C13—C12	111.8 (2)
O1—C3—C2	126.1 (2)	C15—C13—H13	108.6 (17)
N1—C3—C2	110.7 (2)	C14—C13—H13	107.3 (16)
O3—C4—N2	121.7 (2)	C12—C13—H13	108.5 (17)
O3—C4—C5	123.1 (2)	C13—C14—H14A	111.8 (19)
N2—C4—C5	115.2 (2)	C13—C14—H14B	109.8 (18)
C6—C5—C4	106.87 (17)	H14A—C14—H14B	106 (3)
C6—C5—C16	114.2 (2)	C13—C14—H14C	115 (2)
C4—C5—C16	109.36 (19)	H14A—C14—H14C	105 (3)
C6—C5—H5	107.9 (13)	H14B—C14—H14C	109 (3)
C4—C5—H5	110.9 (13)	C13—C15—H15A	109.8 (18)
C16—C5—H5	107.7 (13)	C13—C15—H15B	111.4 (18)
C11—C6—C7	118.1 (2)	H15A—C15—H15B	111 (3)
C11—C6—C5	122.1 (2)	C13—C15—H15C	111.5 (17)
C7—C6—C5	119.8 (2)	H15A—C15—H15C	110 (2)
C8—C7—C6	121.1 (2)	H15B—C15—H15C	103 (2)
C8—C7—H7	121.0 (16)	C5—C16—H16A	110.8 (16)
C6—C7—H7	117.8 (16)	C5—C16—H16B	109.2 (15)
C7—C8—C9	121.1 (2)	H16A—C16—H16B	112 (2)
C7—C8—H8	121.5 (15)	C5—C16—H16C	107.1 (17)
C9—C8—H8	117.4 (15)	H16A—C16—H16C	107 (2)
C10—C9—C8	117.6 (2)	H16B—C16—H16C	111 (2)
			
C3—N1—N2—C4	−77.8 (3)	N2—C4—C5—C16	128.2 (2)
C1—N1—N2—C4	94.6 (2)	C4—C5—C6—C11	−89.8 (3)
N2—N1—C1—O2	6.7 (3)	C16—C5—C6—C11	31.2 (3)
C3—N1—C1—O2	179.2 (2)	C4—C5—C6—C7	87.9 (2)
N2—N1—C1—S1	−174.03 (16)	C16—C5—C6—C7	−151.0 (2)
C3—N1—C1—S1	−1.5 (2)	C11—C6—C7—C8	−0.1 (4)
C2—S1—C1—O2	178.7 (2)	C5—C6—C7—C8	−177.9 (2)
C2—S1—C1—N1	−0.56 (18)	C6—C7—C8—C9	−0.3 (4)
C1—S1—C2—C3	2.22 (19)	C7—C8—C9—C10	0.7 (4)
N2—N1—C3—O1	−4.9 (3)	C7—C8—C9—C12	−177.3 (2)
C1—N1—C3—O1	−177.5 (2)	C8—C9—C10—C11	−0.7 (3)
N2—N1—C3—C2	175.8 (2)	C12—C9—C10—C11	177.3 (2)
C1—N1—C3—C2	3.3 (3)	C9—C10—C11—C6	0.3 (4)
S1—C2—C3—O1	177.4 (2)	C7—C6—C11—C10	0.1 (3)
S1—C2—C3—N1	−3.3 (2)	C5—C6—C11—C10	177.8 (2)
N1—N2—C4—O3	−8.5 (3)	C10—C9—C12—C13	−98.4 (3)
N1—N2—C4—C5	169.96 (18)	C8—C9—C12—C13	79.5 (3)
O3—C4—C5—C6	70.7 (3)	C9—C12—C13—C15	−175.9 (2)
N2—C4—C5—C6	−107.7 (2)	C9—C12—C13—C14	60.8 (3)
O3—C4—C5—C16	−53.4 (3)		

### Hydrogen-bond geometry (Å, °)

**Table:**

*D*—H···*A*	*D*—H	H···*A*	*D*···*A*	*D*—H···*A*
N2—H1N2···O3^i^	0.85 (3)	1.94 (3)	2.713 (2)	151 (2)
C2—H2A···O1^ii^	0.89 (3)	2.53 (3)	3.209 (3)	133 (3)
C2—H2B···O2^iii^	0.92 (3)	2.45 (3)	3.371 (3)	174 (2)
C5—H5···O1^iv^	1.02 (3)	2.46 (2)	3.190 (3)	127.6 (18)
C2—H2A···Cg2^ii^	0.90 (3)	2.99 (3)	3.474 (3)	116 (2)

Symmetry codes: (i) *x*+1/2, −*y*+1/2, −*z*+1; (ii) −*x*, −*y*, −*z*+1; (iii) −*x*+1/2, *y*−1/2, *z*; (iv) −*x*+1/2, *y*+1/2, *z*.
